# Decontamination of multispecies oral biofilm from rough implant surface by airflow with glycine

**DOI:** 10.1002/cre2.507

**Published:** 2021-10-26

**Authors:** Kyla Leung, Jiarui Bi, Georgios Giannelis, Gethin Owen, Hannu Larjava

**Affiliations:** ^1^ Faculty of Dentistry, Department of Oral Biological and Medical Sciences University of British Columbia Vancouver British Columbia Canada

**Keywords:** airflow, biofilm, decontamination, glycine, peri‐implantitis

## Abstract

**Objectives:**

Decontamination of biofilm‐colonized rough implant surfaces remains challenging. We investigated the effect of airflow with glycine powder (AFG) on decontamination of mature oral multispecies biofilm from a sandblasted and acid etched (SLA) titanium surface.

**Materials and Methods:**

Subgingival dental plaque was cultured on SLA disks anaerobically for 21 days. AFG with various settings and distances was applied directly on the disks with or without previous rinse of 0.9% NaCl. The specimens were then analyzed through scanning electron microscope and remaining bacteria on the implant surface were quantified and statistically compared.

**Results:**

Mature oral biofilm with cocci and rods as major morphotypes, as well as spiral‐ and filamentous‐shaped organisms, was formed on the untreated disks. Saline rinsing removed the thick biofilm layer but left numerous of coccoid bacteria in rough surface pits. AFG effectively removed most of the bacteria from the pits. Both 25% and 50% power settings were equally effective at 3‐mm distance. With 50% power, AFG successfully removed bacteria at both 3‐ and 6‐mm distance. When AFG was applied on native biofilm without prior rinsing with saline, it effectively removed the biofilm including bacteria in the pits.

**Conclusion:**

Application of AFG appears effective in removing bacteria from rough implant surfaces.

## 1 INTRODUCTION

The accumulation and incomplete removal of a bacterial biofilm appears to be the etiology of a majority of cases of peri‐implant diseases. Implant microstructure plays a crucial role in biofilm attachment (De et al., 2017). Rough‐surface implants are currently favored over polished‐surface implants due to increased bone to implant contact, cell attachment, biomechanical stability, bone strength, and success rates (De et al., 2017). However, some reports show that increased surface roughness over a threshold of 0.2 microns may encourage more rapid bacterial aggregation to the implant surface than on machined surfaces (John et al., 2015). Once rough‐surface implants are colonized with bacteria, they are exceedingly difficult to disinfect due to lack of access to the implant surface itself (Busscher et al., 2010) and the microscopic irregularities of the surface within which bacteria escape methods for removal (Dostie et al., 2017). Chemical treatments to disinfect rough implant surfaces have yielded poor results with studies showing that bacteria continue to persist following commonly used chemical therapies, such as application of chlorhexidine, tetracycline paste, citric acid, phosphoric acid, sodium hypochlorite, and hydrogen peroxide (Burgers et al., 2012; Dostie et al., 2017; Gosau et al., 2010; Ntrouka et al., 2011). Even when combined with mechanical treatment, chemical disinfection fails to clean the majority of implant surfaces (Alotaibi et al., 2019). Although chemical agents can reduce the biofilm viability, the bacteria quickly regrow to pre‐treatment levels (Han et al., 2019).

Air polishing was introduced in the 1970s as a method for removing stains and bacterial plaque from teeth and implants (Graumann et al., 2013). An airflow device directs a pressurized mixture of air, water and abrasive powder toward the tooth or implant surface to remove stains and biofilm efficiently and effectively in less time than conventional rubber‐cup polishing. Traditionally, sodium bicarbonate powder was used in air polishing (Gutmann, 1998). These powders with particle size up to 250 μm, have not been found to abrade enamel but their extended use may damage dentin and cementum, as well as restorative materials (Graumann et al., 2013). Therefore, less abrasive powders with smaller particle sizes such as calcium carbonate, calcium sodium phosphosilicate and glycine have been introduced to the market as alternatives to sodium bicarbonate (Petersilka, 2011). Several studies have shown efficacy of air abrasives in cleaning implant surfaces with smaller particle size such as glycine causing no damage to the rough implant surface (Wei et al., 2017). Therefore, airflow with glycine (AFG) is of particular interest with respect to implant surface disinfection with many studies supporting its use in conjunction with other methods of managing and treating peri‐implant diseases (Menini et al., 2019; Schwarz et al., 2015; Tastepe et al., 2012). A majority of available studies assessing the efficacy of AFG on implant surface cleaning have only assessed its ability to remove stains, ink, specific bacteria and bacterial endotoxins. As such, there is a lack of studies specifically assessing the efficacy of the disinfection of a mature, multispecies biofilm on a rough‐surface implant using AFG.

The aim of the present study was to assess whether AFG in different power settings and from different distance would be efficacious in the disinfection of a mature oral biofilm from sand‐blasted and acid etched (SLA) titanium implant surface.

## 2 MATERIALS AND METHODS

### 2.1 Cultivation of mature biofilm on sandblasted and acid etched disks

Sterile SLA® implant disks measuring 5 mm in diameter and 1 mm thick (Straumann®) were rinsed with phosphate‐buffered saline (PBS; Sigma‐Aldrich) and coated with bovine dermal type I collagen (10 μg/ml collagen in 0.012 M HCl in water; Cohesion) overnight at 4°C. Subgingival plaque samples were obtained from maxillary molars of a single systemically healthy volunteer with relatively healthy periodontium except minor gingivitis using toothpicks (University of British Columbia ethics protocol #H15‐01881). An aliquot of bacterial suspension was placed in each well (approximately 3.0 × 10^7^ CFU/ml) in 2 ml of brain heart infusion broth (Becton‐ Dickinson) as previously described (Bi et al., 2016; Bi et al., 2017; Schuldt et al., 2020). The discs were incubated under anaerobic conditions (AnaeroGen; Oxoid) at 37°C for 21 days changing medium once a week.

### 2.2 Treatment protocols

Following the completion of the incubation period, the biofilm‐contaminated discs were placed in triplicates into different treatment regimens or control group. Each treatment regimen was then repeated three different times using three different biofilms from the same donor (Bi et al., 2017). The control groups included a no‐rinse control with an uninterrupted biofilm (*n* = 6), a single‐rinse control (*n* = 6) in which the contaminated discs were rinsed once with 6 ml of sterile saline (1 ml increments) and a double‐rinse control (*n* = 6) in which the contaminated discs were rinsed twice with 6 ml 0.9% sterile saline. For testing of AFG, the Air‐Flow Master Piezon® (EMS SA, CH‐1260 Nyon) was used with subgingival nozzle. Using this device, glycine contained within a separate, dry canister would be directed through the nozzle and mixed with pressurized water to create the slurry of water and glycine. AFG was applied either at 50% (21–25.4 psi) or 25% power setting (10.5–12.7 psi) at 3‐ or 6‐mm distance from the disk (*n* = 6–9 each group). Most of the experiments were done on disks that were previously rinsed with saline mimicking AFG application in clinical setting. In a set of experiments, however, AFG was applied directly on biofilm disks at 3 mm distance without any prior rinsing (*n* = 6). We also tested the AFG without the powder in a similar setting (*n* = 6). All the disks were rinsed with saline after the AFG treatments.

### 2.3 Processing of samples for scanning electron microscopy and the data analysis

The disks were processed for scanning electron microscopy (SEM) as previously described (Dostie et al., 2017; Schuldt et al., 2020). Imaging of each sample for assessment was done under SEM (Helios Nanolab 650 Focussed Ion Beam SEM). Three random SEM images were taken at the center of each disk at a voltage of 1 kV and at a magnification of 5000× times. The field of view in each detailed image was 30 μm by 20 μm and represented a surface area of 600 μm^2^, approximately 3% of the total surface area of each disk. The number of bacterial cells in each field was counted and averaged using ImageJ 1.51m9 software (National Institute of Health). Therefore, each disk had one single number representing average number of bacteria per field on that disk. Replicate disks were used to calculate the mean ± SD and ± SEM.

### 2.4 Statistical analysis

Statistical analysis was done using a two‐way analysis of variance and post hoc Tukey test using data collected from the mean bacterial counts pooled from each group. Only *p* values with a *p* < 0.05 were considered significant.

## 3 RESULTS

### 3.1 Removal of mature multispecies biofilm by rinsing with physiological saline

**Figure 1:**
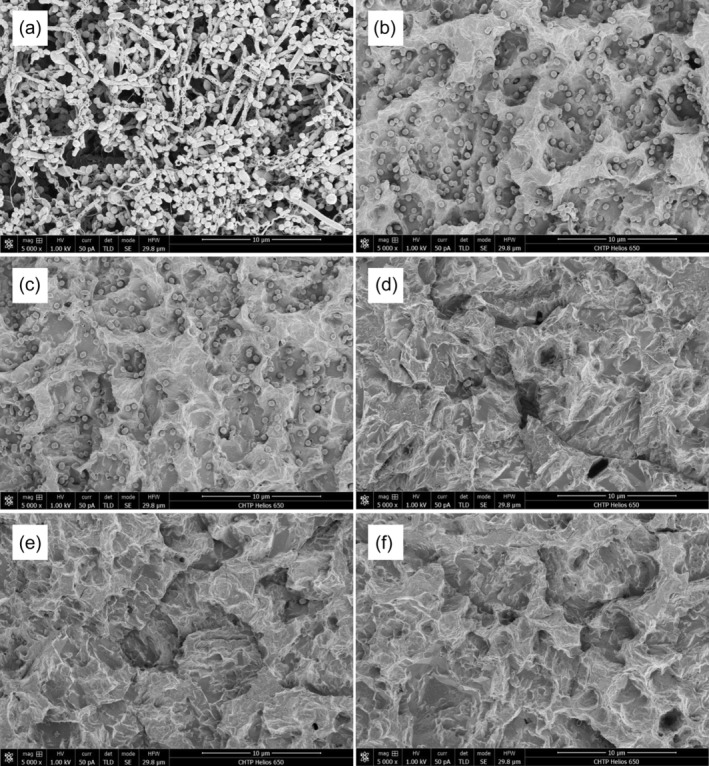
Effect of air flow with glycine (AFG) on bacteria on rough implant surface. (a) untreated mature oral biofilm grown for 3 weeks on sandblasted and acid etched surface as visualized on scanning electron microscopy (SEM) at 5000× magnification. (b) Residual bacteria (mainly cocci) present after single‐rinse (6 ml saline in 1 ml increments) control (SEM 5000×). (c) Residual bacteria after double‐rinsing the biofilm (12 ml in 1 ml increments; 5000×). (d) Implant surface after AFG (50% power) at distance of 3 mm from surface (5000×). (e) Implant surface after AFG (50% power) at a distance of 6 mm from surface (5000×). (f) implant surface after AFG (25% power) at distance of 3 mm from surface (5000×)

**Figure 2:**
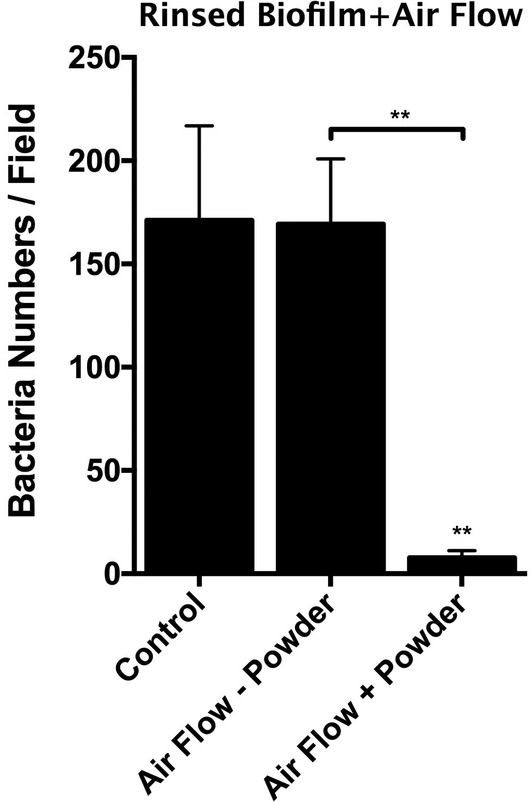
Quantification of residual bacteria (bacteria numbers per field) after double rinsing and airflow with and without glycine powder at 3 mm distance. Air flow with glycine shows statistically significant reduction in bacterial cells on the SLA surface (**p < 0.01). Number of bacteria in undisturbed intact biofilm cannot be enumerated due to high numbers estimated to be around 6 × 10^7^ per disk

**Figure 3:**
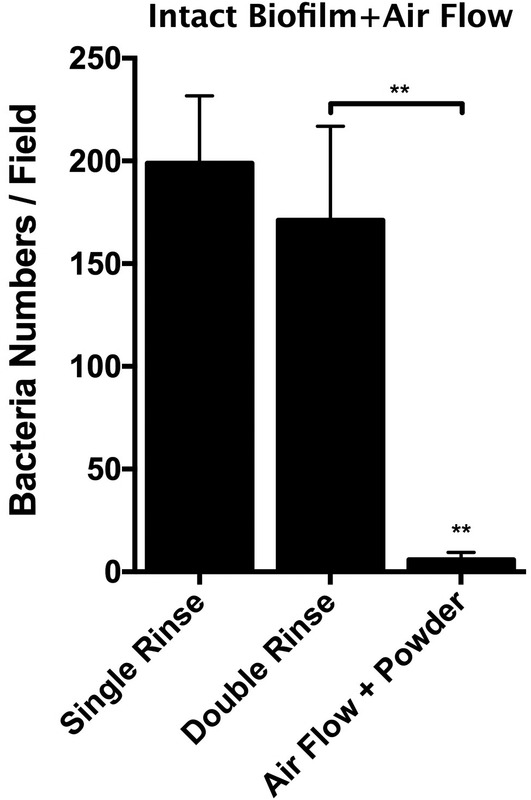
Quantification of residual bacteria (bacteria numbers per field) after single rinse, double rinse and air flow with glycine (AFG) on intact biofilm at 3 mm distance. AFG shows statistically significant reduction of bacterial cells on the sandblasted and acid etched surface (**p < 0.01). Number of bacteria in undisturbed intact biofilm cannot be enumerated due to high numbers estimated to be around 6 × 10^7^ per disk

Following 3‐week incubation period, mature, multispecies biofilm was present on the SLA® disks, completely obscuring the SLA® surfaces and resembling oral biofilms found in vivo (Figure 1a). The majority of bacteria were cocci which appeared to aggregate together in clusters and with rods, sometimes resembling corncob formations, similar to the conformations of bacteria in mature in vivo plaque. Spiral‐ and filamentous‐shaped organisms and spirochetes were also observed but with less frequency compared to rods and cocci. With various layers of bacterial cells superimposed, imaging of deeper layers of bacteria was evidently obscured. The number of bacteria observed was too large to accurately enumerate. Most of the specimens were rinsed with 6 ml of saline solution in 1 ml increments before any other treatment was applied to mimic clinical setting. This single saline rinsing (single rinse) alone removed majority of the biofilm, leaving behind the deepest layer of cocci‐shaped bacteria that remained attached to the SLA® surface within the grooves and pits (Figure 1b). Since all specimens with AFG treatment were rinsed again afterwards with saline solution to remove any debris from the powder, we also included control specimens that had received a second rinse (double‐rinse controls). The second round of rinsing (double rinse) did not remove significantly more bacteria compared to single rinse (*p* > 0.05; Figures 1c and 2). The mean bacterial counts per field in single‐rinsed and double‐rinsed controls were 198.9 and 171.1 cells, respectively (Figure 3).

### 3.2 Removal of mature multispecies biofilm with air flow with glycine

In the first set of experiments, we applied AFG on biofilm that had been previously rinsed by saline solution. The use of AFG (50% power, 3 mm distance) successfully removed the majority of the biofilm, including the coccoid microorganisms from the pits of the SLA surface that were not removed by saline rinsing alone (Figure 1d; Figure 2). Small number of cocci could still be found scattered in pits and irregularities of the surfaces. The mean bacterial count after treatment with AFG was 7.7 cells per field of view. Airflow without glycine powder was also tested and found to be no more effective in decontamination of the SLA surfaces than the saline rinse control (*p* > 0.05; Figure 2). The mean bacterial count when samples were treated with airflow alone was 169.3 cells/field (Figure 2). Next, we tested whether AFG could eliminate the mature, multispecies biofilm without pre‐rinsing with saline. Compared to saline rinsing (single or double), AFG effectively removed the intact biofilm and even the bacteria hiding in the pits (Figure 3). The mean bacterial count after treatment the undisturbed mature biofilm with AFG was 5.9 cells per field of view (Figure 3).

### 3.3 Removal of mature multispecies biofilm with air flow with glycine at different settings

**Figure 4:**
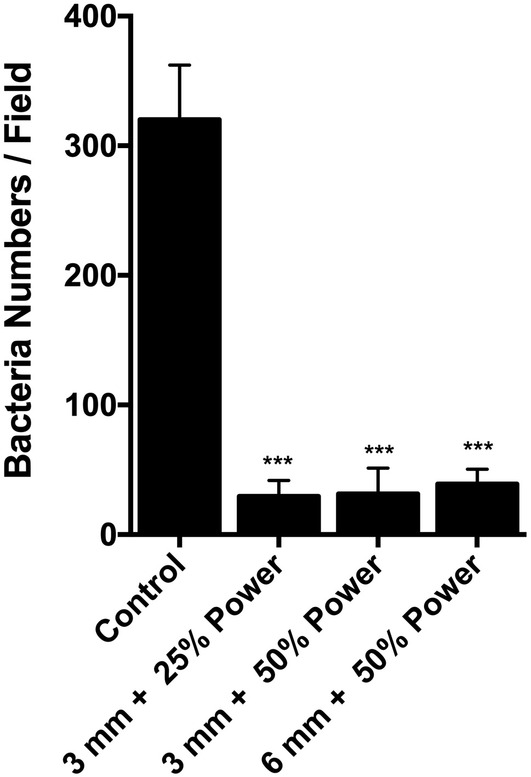
Quantification of residual bacteria (bacteria numbers per field) after saline rinsing (control), air flow with glycine (AFG) at 3 mm distance with 25% power, at 3 mm distance with 50% power and at 6 mm distance with 50% power. AFG in all settings shows significant reduction of bacterial cells on the sandblasted and acid etched surface (***p < 0.0001). Number of bacteria in undisturbed intact biofilm cannot be enumerated due to high numbers estimated to be around 6 × 10^7^ per disk

In clinical setting, the distance of the tip of the AFG handpiece cannot be always kept in stabile 3 mm distance from the implant surface. Therefore, we tested different distances at the standard power setting of 50%. Increasing the distance of the tip from 3 to 6 mm did not reduce the effectiveness of the AFG in removing the biofilm from the pits of the SLA surface (*p* > 0.05; Figures 1e, 4). We also tested whether reducing the power from 50% to 25% would change the efficacy of the AFG treatment. The results showed that both power settings were equally effective removing the biofilm from the pits of the SLA surface (*p* > 0.05; Figures 1f, 4).

## 4 DISCUSSION

This study clearly demonstrated the effectiveness of AFG in biofilm decontamination from rough implant surface. Rinsing prior to the AFG made little to no difference in the effectiveness of AFG in decontamination of the implant surface. Airflow without glycine proved to be ineffective with respect to removing the biofilm compared to AFG. It is difficult to reconcile the average size of glycine particle (45 μm) and that of the roughened lacunae and grooves on the surface which measured approximately 10 μm. It may be possible that glycine, being water soluble, will begin to dissolve when added to the water within the airflow device creating variable, smaller sizes particles which may access the smaller grooves of the rough surface implant.

The number of microbes in the untreated biofilm was far too great to accurately enumerate. In all the treatment groups, the majority of the biofilm was removed. In the control groups, however, continued presence of microorganisms in the pits and grooves of the implant surface was a common finding. In comparison to similar studies (Dostie et al., 2017) using the same biofilm model, AFG proved to be much more effective in removing bacteria from the rough pits and grooves than chemical disinfection. In addition, because glycine has an inhibitory effect on bacterial growth (Cosquer et al., 2004; Holo & Nes, 1989), its presence following treatment may result in a sustained antibacterial effect on the implant surface (Cochis et al., 2013).

The morphology of the persisting bacterial cells is also of interest. In the untreated biofilm, it is evident that a multispecies, stratified biofilm is present, representative of what might be expected in peri‐implant diseases (Sanz‐Martin et al., 2017). Morphologies that are visible include filamentous rods, spirochetes and coccoid cells organized in layers in a thick, three‐dimensional structure. Following the removal of a majority of the biofilm with sterile saline rinsing only cocci with occasional rods remained. These bacteria appear to have the same morphology as the early colonizers, suggesting that AFG is successful in removing the majority of the biofilm, including many of the base layer of microbes which directly attach to the implant substratum (Teles et al., 2012).

According to recommendations for use, our particular AFG device was directed at a 45° angle to the tooth or implant surface at a distance of 3 mm from the surface. It was also recommended that the power settings be set to approximately 50%, although the manufacturer's recommendations do instruct to begin with powder settings at a minimum before increasing steadily as required when used clinically. According to the manufacturer, the maximum dynamic pressure produced by the AirFlow Master Piezon when set to “PERIO” mode ranges from 42–50.8 psi. Therefore, at a power setting of 25%, the pressure generated by the unit would range from 10.5 to 12.7 psi, and at a power setting of 50%, the pressure generated would range from 21 to 25.4 psi. In our study, we selected distances of 3 or 6 mm to assess the effect of distance on the efficacy of biofilm removal. The ability of AFG to remove the biofilm from the implant surface was unchanged regardless of power setting or distance from the surface to the nozzle. Indeed, there was no statistically significant difference between the results of AFG under different settings. These results contradict previous findings demonstrating that increasing pressure of airflow with abrasive powders would increase the efficacy of cleaning. In this particular study, ink was used to stain the surface and cleaning efficacy was measured by ink removal alone (Wei et al., 2017). AFG was found to be relatively ineffective at removing ink at a 25 psi, but increased in efficacy at 35 psi (Wei et al., 2017). Effects for all test groups appeared to plateau at a certain air pressure and while higher pressures appeared to result in better cleaning efficacy, more surface modification was noted, even in the glycine‐treated groups (Wei et al., 2017), an observation that was not made in our particular study.

It is not feasible in vivo that a distance of 3 mm can always been maintained between the AFG source and the implant surface, especially when one considers the high likelihood of obstruction by adjacent teeth, restorations, implants, and/or the superstructure of the implant. Indeed, even the depth of the peri‐implant pocket would serve as a factor in increasing distance between the airflow source and the implant surface. As such, 6 mm was selected as the upper range of distance between the device and the biofilm and therefore might best replicate the distance between the implant surface and AFG in vivo.

In this study, the biocompatibility of the surface and the healing properties, if any, of glycine were not assessed. Studies have assessed the use of biocompatible, osteoconductive powders with air‐polishing on contaminated titanium surfaces and have found promising results with respect to cell differentiation on treated surfaces (Tastepe et al., 2012). Other studies have well established that AFG alters little of the physical and chemical surface characteristics (Keim et al., 2019; Lollobrigida et al., 2020). Future directions in our study might be to include the assessment of fibroblast and/or osteoblast cell attachment, growth and differentiation when seeded onto AFG‐treated implant surfaces.

The intra‐surgical use of AFG is not yet been approved by the Federal Food and Drug Administration or Health Canada. Indeed, the risk of air emphysema, although small, if used non‐surgically or surgically exists (Alonso et al., 2017; Bassetti et al., 2014; Lee et al., 2018; Schwarz et al., 2016). Only two cases of emphysema related to peri‐implantitis treatment and one to peri‐implant cleaning have been reported in the literature spanning 1987–2018 (Alonso et al., 2017). The other cases (*n* = 6) have been linked to stain, plaque or calculus removal from teeth (Alonso et al., 2017). There are no reports describing emphysema after surgical peri‐implantitis treatment using air power abrasive devices. However, based on the premise of this study, in order to adequately remove the biofilm from the implant surface using AFG, the clinician may be required to gain direct access to the implant surface to avoid obstruction of the implant surface. Indeed, in order for AFG to have much effect, it appears that surgical access to subgingival implant surfaces would be required. The macrostructure of the implant also presents a challenge with respect to access. Due to the threading on dental implants, the pitches of the implants may remain inaccessible for the most part should the AFG be directed from coronal to apical as would be the case in a nonsurgical approach. However, it would be reasonable to assume that in order to achieve effective decontamination of the implant in vivo, the clinician may be required to angulate the AFG nozzle above and under the threads in order to access the entirety of the pitches. As such, for purposes of managing and treating peri‐implantitis, it may be required that AFG be used intra‐surgically. Because of the use of air in this device, other devices without inclusion of air should be tested for implant decontamination.

With respect to the manner by which AFG actually decontaminates the implant surface, it is unlikely that the antimicrobial properties of glycine, which a number of studies have alluded to (Cochis et al., 2013; Cosquer et al., 2004; Holo & Nes, 1989), play much of a role in eradicating the biofilm. Based on the short duration of the AFG, it is unlikely that there is the exposure time of the biofilm to the glycine is long enough for the antimicrobial effects to take place. Instead, it is more likely that the abrasive qualities of the powder itself serve to physically remove the biofilm as opposed to chemically.

There are several limitations in the present study. Because of in vitro design, the results may not reflect clinical effectiveness of AFG due to biofilm composition and adhesion that is likely to be different in vivo compared to cultivated multispecies biofilm used for the study. In addition, the SLA disks lack the macrostructure of dental implants making them easier to disinfect. Furthermore, application of the AFG including nozzle angulation is much more complicated in a clinical setting. Future studies should address native biofilm removal on extracted implants that possess different micro‐ and macro‐structured surfaces.

## 5 CONCLUSION

The use of airflow with glycine is significantly more effective in the removal of a mature, multispecies biofilm from a roughened titanium surface than control treatments with sterile saline rinsing alone. Glycine particles appeared to be necessary to the effectiveness of air flow. No significant differences in the effectiveness of AFG were found between variable power settings of the AFG unit and distances between the AFG nozzle and biofilm.

## AUTHOR CONTRIBUTIONS

Hannu Larjava conceptualized and supervised the project. Jiarui Bi, Gethin Owen, and Hannu Larjava contributed in methodology. Jiarui Bi, Gethin Owen helped in software application instead of development. Kyla Leung, Jiarui Bi, and Gethin Owen contributed in data collection and data analysis. Kyla Leung, Jiarui Bi and Hannu Larjava helped in writing the original draft, whereas, Jiarui Bi, Georgios Giannelis, Gethin Owen and Hannu Larjava helped in review and editing. Hannu Larjava helped in project administration and funding acquisition. All authors read and approved the final manuscript.

## CONFLICT OF INTEREST

The authors declare no conflict of interest.

## Data Availability

The data that support the findings of this study are available from the corresponding author upon reasonable request.
